# Interventions to Improve the Quality of Life of Patients with Chronic Obstructive Pulmonary Disease: A Global Mapping During 1990–2018

**DOI:** 10.3390/ijerph17093089

**Published:** 2020-04-29

**Authors:** Giap Van Vu, Giang Hai Ha, Cuong Tat Nguyen, Giang Thu Vu, Hai Quang Pham, Carl A. Latkin, Bach Xuan Tran, Roger C. M. Ho, Cyrus S. H. Ho

**Affiliations:** 1 Department of Internal Medicine, Hanoi Medical University, Hanoi 100000, Vietnam; 2 Respiratory Center, Bach Mai Hospital, Hanoi 100000, Vietnam; 3 Institute for Global Health Innovations, Duy Tan University, Da Nang 550000, Vietnam; 4 Faculty of Pharmacy, Duy Tan University, Da Nang 550000, Vietnam; 5 Faculty of Medicine, Duy Tan University, Da Nang 550000, Vietnam; 6 Center of Excellence in Evidence-based Medicine, Nguyen Tat Thanh University, Ho Chi Minh City 700000, Vietnam; 7 Institute for Preventive Medicine and Public Health, Hanoi Medical University, Hanoi 100000, Vietnam; 8 Bloomberg School of Public Health, Johns Hopkins University, Baltimore, MD 21205, USA; 9 Institute for Health Innovation and Technology (iHealthtech), National University of Singapore, Singapore 119077, Singapore; 10 Department of Psychological Medicine, Yong Loo Lin School of Medicine, National University of Singapore, Singapore 119228, Singapore; 11 Center of Excellence in Behavioral Medicine, Nguyen Tat Thanh University, Ho Chi Minh City 700000, Vietnam; 12 Department of Psychological Medicine, National University Hospital, Singapore 119074, Singapore

**Keywords:** scientometrics, content analysis, text mining, interventions, COPD, QoL

## Abstract

Chronic obstructive pulmonary disease (COPD) has been considered a significant health challenge globally in recent years, which affects different aspects of the quality-of-life (QoL). A review was conducted of research output, research topics, and landscape to have a global view of the papers mentioning the interventions to increase QoL of patients with COPD. A total of 3242 research items from Web of Science during the period 1990–2018 were downloaded and analyzed. Analyses based on the different levels of data and methods using using VOSviewer software tool (version 1.16.15, Centre for Science and Technology Studies (CWTS), Leiden University, Leiden, The Netherlands) and Latent Dirichlet allocation. By exploring the trends in research productivity and topics, an increase was found in the number of papers mentioning non-pharmacological interventions as well as mental health illness and QoL among patients with COPD. In conclusion, the research on the interventions to increase the QoL of patients with COPD has attracted scientists globally. It is suggested that more research should be conducted on the effectiveness of non-pharmacological therapies to increase QoL of patients with COPD that can be applied broadly in the community. The collaboration and support from developed countries to developing countries are needed to increase the QoL of people living with COPD.

## 1. Introduction

Chronic obstructive pulmonary disease (COPD) is one of the chronic airway diseases, which characterized by the limitation in airflow and not fully reversible [1]. The reported prevalence of COPD is different among regions: 4% in Europe [2], 6.3% in the Asia Pacific region [3], from less than 4% to over 9% [4] in the US and predicted, with limited epidemiological evidence, to be at about 11% in 2010 in the African region [5,6].

This chronic disease has significant adverse effects on physical and mental conditions of those patients [7,8,9], as other systems and organs other than the lungs suffered the negative impacts, leading to pneumonia [10], pulmonary hypertension [11], and cardiovascular disease (CVD) [12]. A worsening mental status has been found in patients with COPD compared to non-COPD subjects, with higher rates of anxiety and depression [13,14], and the severity of fatigue [15]. Patients with COPD less frequently report a partner compared to others, and, when having a partner, they were less likely to be ‘very satisfied’ with the daily support and less often perceived emotional support from the partner [16]. They suffer worse quality of life with early-morning and nighttime symptoms compared to those without COPD [17,18]. COPD often results in a reduction in quality of life. 

Quality of life (QoL) is defined by World Health Organization as a broad and complex concept of an individual about their physical health, mental health, social relationships and beliefs in the context of their living enviroment [19]. It is a “multidimensional measure” which focus on at least three domains: physics, psychology and society. Thus, several studies applied dimensions of QoL to to: (1) evaluate the efficiency of clinical therapies [20,21,22] or alternative therapies [23,24]; (2) identify factors associated with QoL [25] and increase health service quality [24]. In the case of COPD, improving QoL of patients with COPD becomes critical due to the incurables [26]. Medical methods have been used mainly to control COPD and strengthen prevention efforts, such as: (1) smoking cessation [27,28], (2) pharmacotherapy [29], and (3) Non-pharmacological therapy [30]. Therefore, measuring QoL could be useful in applying suitable interventions and preventing risk factors affected COPD. 

Systematic reviews of interventions and treatments are considered as a reliable source of evidence to inform clinical practice and policy development [31]. Several systematic reviews and meta-analyses mentioning interventions to patients with COPD have been conducted. Gregersen et al. confirmed that telehealth showed promise for improving QoL of patients living with COPD, yet, this method call for more research to prove its effectiveness [32]. According to Coronini-Cronberg et al., psychosocial and pharmacological support is an effective intervention for smoking cessation [33], which raises the QoL in some health domains [34]. Moreover, breathing exercises can be used to improve the QoL of patients living with COPD, yet, the use of this method as a complementary therapy needs more research [35]. These literature review studies answer specific questions by gathering available empirical research evidence. However, a limitation of this approach is that it could review one method, which makes it difficult to compare the effects of all methods during a long period of study.

In addition, several researchers have used indicators of scientometric to review literature [36]. Prvevious studies used bibliometrics to explore research output, country collaboration, journal ranking or fundings of all papers mentioning COPD in European countries [37] or in Arab countries [38]. However, scientometric analyses may not have a deep understanding of the context of research or the landscape of research areas. Therefore, by combining scientometric and Latent Direcht allocation, topic modeling (in titles and abstracts), this study aims to describe the global trend in research outputs, countries collaboration, interdisciplinary research areas, as well as ten common topics among papers mentioning interventions to improve QoL of patients with COPD. The findings will emphasize research gaps, and make it possible to recommend some implications for future studies and policy. 

## 2. Materials and Methods 

### 2.1. Database and Search Strategy

The data were retrieved in the middle of 2019 from the Web of Science (WoS) Core Collection. It was decided to choose WoS because WoS (1) allows to download a large number of papers and (2) provides necessary information for scientometrics analysis, such as authors’ affiliations, authors’ keywords, the title of papers, publication year, research areas, as well as the number of citations and download times for each paper [39,40].

**Figure A1:**
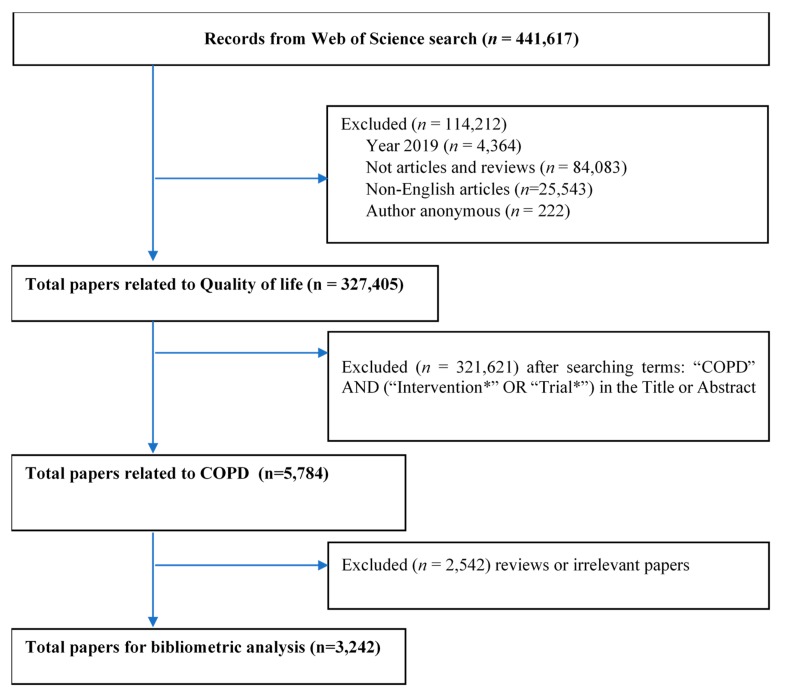
Selection of papers.

The search strategy was described as follow:

- Step 1: With the use of Boolean operators “OR”, the search query was developed to identify the number of published items related to “Quality of life” OR “well-being”. Only English research articles and research reviews were included, while grey literature, conference proceedings, or books/book chapters in any other language were excluded. Papers having anonymous authors and publications in 2019 were also limited. This research began in the middle of 2019; thus, this data could not reflect the research trend for the whole year. Data in WoS databases under text format was downloaded and imported to STATA version 15.0 (STATACorp., Texas, TX, USA) for further extraction. (See Table A1)
- Step 2: STATA syntax was applied to filter the papers in step 1 with the terms “Intervention” OR “Interventions” OR “trial” OR “trials” in titles or abstracts.
- Step 3: The COPD keywords were formed by COPD specialists and reviewing some papers and MeSH term library of PubMed. These terms were used to search in the title and keyword fields among papers in step 2 (see Table A1), and there were 5784 papers for further screening.
- Step 4: Two researchers separately screened the titles and abstracts of 5784 papers to exclude papers not related to COPD. A group discussion with a senior researcher was conducted if there were any contradictions. A total of 3242 papers were imported to STATA for further analysis. (See Figure A1).

### 2.2. Data Analysis

The corrected data after the screening was imported to STATA for further analysis using the following information of the articles: authors’ affiliations, the title of papers, the journals’ name, authors’ keywords, the number of citations, research areas, and abstracts.

Several basic characteristics of the data sets were included publication year, the number of papers /per year, total citations up to 2018, average citation rate per year, total number of downloads in the last six months/five years, and average number of downloads (mean use rate) the last six months/five years. Two network graphs showing the countries collaboration and co-occurrence terms in title and abstracts were established by VOSviewer (version 1.16.15, Centre for Science and Technology Studies (CWTS), Leiden University, Leiden, The Netherlands). Latent dirichlet allocation (LDA) was used for classifying papers into topics [41,42,43,44,45]. The titles and abstracts of most cited papers within each group were reviewed. After discussing with COPD specialists, the labels for each topic were named. In addition to the number and percentage of publications of each topic, these topics were ranked based on the total number of publications in the past five years to explore the research interests. Table 1 shows the methods and results for each kind of data.

## 3. Results

### Overall Growth and Essential Characteristics of Research

Table 2 described the basic characteristics of publications. The first seven papers related to this health issues in dataset were published in 1991. There has been a gradual raise in the annual number of papers on intervention to improve the QoL of patients with COPD within the period 1991–2018, contributing to a total of 3242 papers. The papers in 2018 showed the reading interests of readers in last six month with the average times of download (mean use rate) was 1.8; meanwhile, the papers in 2013 received the highest concern in last five years with average times of download (the mean use rate) was 2.1. The papers in the year 2000 had the highest average citation with 6.9 citations per paper.

The paper having the highest influence was the second report entitled Global Strategy for the Diagnosis, Management, and Prevention of COPD published in 2007 with 3456 citations [46].

**Figure 1:**
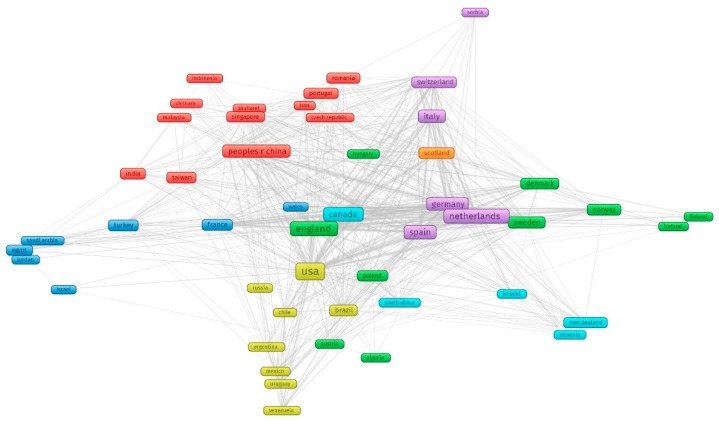
Countries collaboration network. Note: four main clusters, including (1) red cluster: Asia countries and two East European countries (Czech and Romania); (2) yellow cluster: the U.S and South American countries; (3) turquoise cluster Canada and South Africa, New Zealand and European countries; (4) the rest: European countries with three subgroups with the lead of France, the Netherlands and England.

Figure 1 shows countries collaboration network. In total, there were 89 countries contributing for the research field (automatically calculated by VOSviewer). In figure there were 64 countries with minimum of 5 papers. Of those, the United States of America led in the number of studies with 786 papers (24.2%), followed by England (452 papers, 13.9%), the Netherlands (322 papers, 9.9%), and Canada (268 papers, 8.3%). Although people living in low-and middle-income countries (LMICs) are more vulnerable to developing COPD [47], there was only China in the list of top 10 countries having the highest volume. As can ben seen, there were four main clusters in this countries network (1) Asia with the leadership of China in collaboration with two East European countries (Czech and Romania) (red cluster); (2) the U.S and South American countries (yellow cluster); (3) Canada, South Africa, New Zealandm and European countries (turquoise cluster); (4) European countries with three subgroups with the lead of France, the Netherlands, and England (the rest).

**Figure 2:**
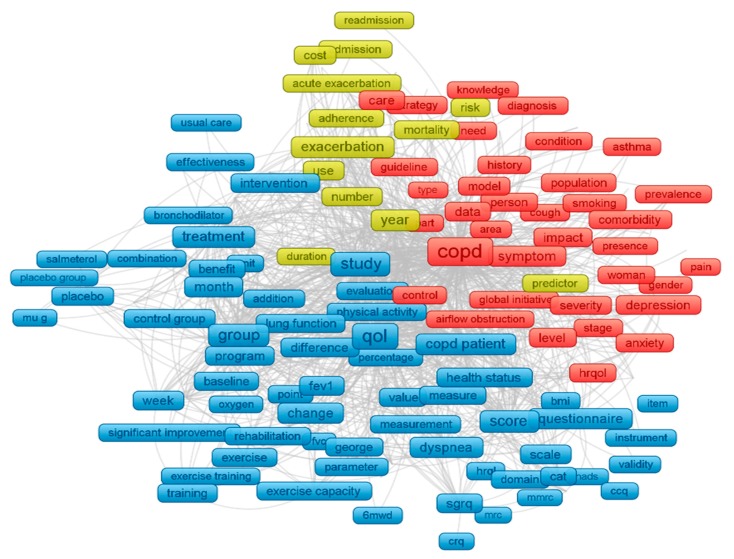
Text mining using VOSviewer (titles and abstracts). Note: the colors of each node were automatically assigned by VOSviewer based on its score; the node size was based on the frequency of each term; the length and thickness of the lines reflected the association between two terms. Cluster 1 (red) refers to comorbidity and COPD; cluster 2 (blue) focuses on interventions and treatment to increase QoL of people with COPD, cluster 3 (yellow) points out the risk and mortality of exacerbation of COPD.

By analyzing abstracts and titles, the most co-occurrence terms were found to discover the scope of COPD research (Figure 2). Three major clusters were formed by 279 most common terms in title and abstract with the minimum appearance of 95 times. The three significant clusters are: Cluster 1 (red) refers to comorbidity and COPD, among which mental health illness (depression and anxiety) was most frequently mentioned. Cluster 2 (blue) focuses on interventions and treatment to increase QoL of people with COPD. Cluster 3 (yellow) points out the risk and mortality of exacerbation of COPD.

Table 3 shows the most cited papers. Each had more than 100 citations during the study period. Based on the list, three main topics which have been recently attracted the attention of researchers were: (1) The Global Initiative for chronic obstructive lung disease (GOLD) reports and other national reports. GOLD was a consensus report published periodically since 2001. It included the latest evidence for diagnosis and prevention from experts, which were as “strategy documents” for adequate care for COPD at a global level [48] (paper 1, paper 7, paper 4, paper 9, paper 28, paper 36 ); (2) Exacerbations in patients with COPD (paper 2, paper 3, paper 12, paper 25, paper 29, paper 32); (3) Treatments and interventions of COPD (paper 5, paper 6, paper 11, paper 14, paper 15, paper 17, paper 19, paper 20, paper 23, paper 24, paper 27, paper 33, paper 34, paper 35, paper 39, paper 40), (4) QoL, health-related QoL and COPD (paper 26, paper 30, paper 31, and paper 41), others topic (rehabilitation—paper 8; COPD and comorbidity—paper 10; COPD and its effects to patient health and life—paper 22 and paper 37).

Applying latent dirichlet allocation in title and abstracts, ten major research topics were formed (Table 4). Topic 2 (*n* = 468 papers), Topic 1 (*n* = 436 papers), and topic 3 (*n* = 355 papers) were three topics with the highest volume of publications. Pulmonary rehabilitation has been a rapidly developed field in the last decades [49]. Further, improving QoL of patients living with COPD by pharmacological therapies (topic 6) or non-pharmacological therapies (topic 3, topic 10) has been a major area of focus. Notably, the domain of mental health received frequent attention from the scientific community with 436 papers. The reason for it could be that about 85% of people living with COPD were at high risk of developing anxiety disorders compared with healthy people [50].

**Figure 3:**
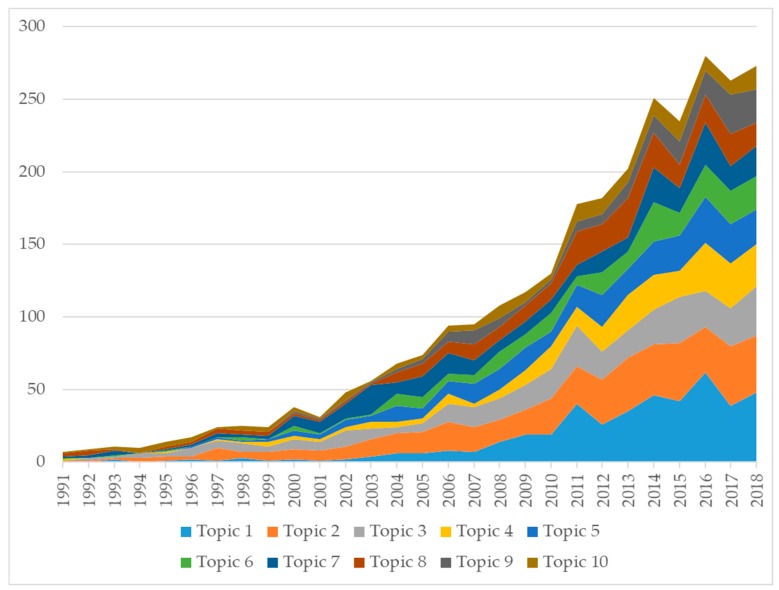
Changes in research topics development.

Figure 3 shows the changes in the development of topics. Topic 1 in the last five years (2014–2018) had the highest number of published papers (*n* = 237), followed by topic 2 (*n* = 186) and topic 3 (*n* = 141).

**Figure 4:**
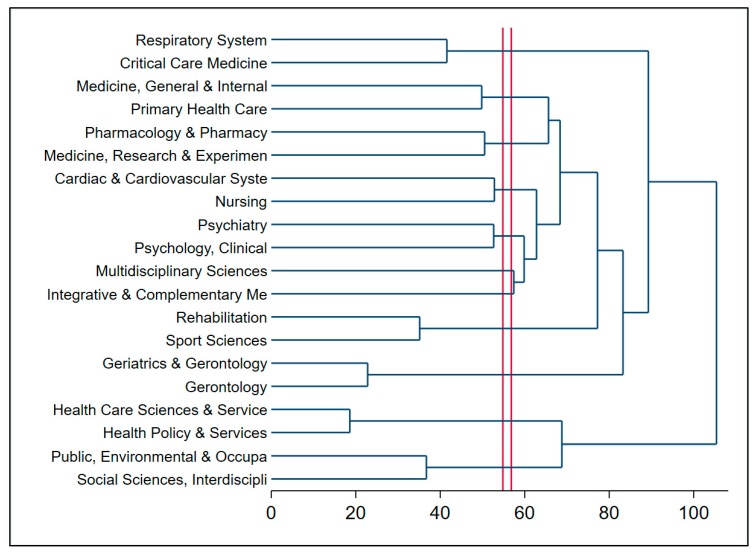
Dendrogram of coincidence of research areas.

Figure 4 shows the cluster of research areas in QoL of patients with COPD. The horizontal axis represents the distance between clusters, while the vertical axis displays the research areas [51]. The red lines show the depth for the cut-off of the analysis [52]. Research landscapes were divided into three main parts. The root (first group) in the top of the dendrogram included (a) respiratory system and (b) critical care medicine. This cluster had a close relationship with (1) intervention and health care such as general & internal medicine, pharmacy, nursing, and cardiovascular system (second group); (2) comorbidities, for instance, psychiatry, clinical psychology, clinical neurology (third group). However, the first group did not have a strong relatedness to the cluster in the bottom, such as rehabiliation or the integration of public health, environmental and occupational health (health care science & services; health policy & services, occupational &public, environment, interdiscriplinary social sciences).

## 4. Discussion

This study investigated the global trend of 3242 research publications regarding interventions to increase QoL of patients with COPD. It was found that the publications of research related to in topic increased annually and gradually, and most of the contribution came from high income countries (HICs). Mental health issues and non-pharmacological therapy, including exercise, home care, self-care education, noninvasive ventilation, and oxygen therapy were common approaches. Current findings emphasize the importance of research that focuses on the effects of non-pharmacological therapy, which should be considered to increase QoL of people living with COPD. Additionally, mental health problems among people living with COPD have received more focus, especially in the last five years. 

Notably, but unsurprisingly, a high number of research were conducted by authors from HICs than that of LMICs although more than 90% of COPD-related deaths occur in LMICs [53]. This work supports the conclusion of previous studies, which confirmed the main contribution of HICs in diabetes research [54] or HIV/AIDS research [55]. This phenomenon may be explained by the fact that risk factor prevention has not been fully recognized by the LMICs’ governments and populations, including using biomass fuels indoors for cooking [56] or occupational exposure [57]. Moreover, many LMICs faced the barriers in research and implication planning, such as information and communication technology limitations [58], lack of human resources and finance, and scientific findings [59]. Therefore, the support of HICs and actively joining in collaboration network with HICs are critical to LMICs [60].

Chronic obstructive pulmonary disease is a chronic disorder, which requires a long-term treatment with complementary and alternative therapy to reduce exacerbation and improve patients’ QoL [61]. Our finding were in line with the results of previous studies, which emphasized that pharmacological therapy [62], exercise [63], non-invasive ventilation [64], and oxygen therapy [65] increase the QoL of people living with COPD. In our study, the number of papers mentioning pharmacological treatment was in the top five of highest volume of work by LDA. It showed the concern of researchers and physicians on this therapy to control the symptoms in stable COPD as well as improve QoL of people suffered COPD. It confirmed the results of some papers which emphasize the effectiveness of pharmacotherapy in controlling symptoms to decrease recurrence and seriousness of exacerbations and improve QoL [66,67]. However, this topic rose at a lower level in the last five years compared with non-pharmacy therapies, such as mental health, or rehabilita. The results might be explained by the efficiency of the alternative therapies in improving the quality of life, controlling symptoms in daily life and when exacerbations occur [68], and reducing the frequency of hospitalization [69].

Furthermore, the topics receiving the most attention in the last five years were comorbidities and mental health issues in patients with COPD. A previous study showed that about one-third of patients living with COPD with depression or anxiety did not received appropriate treatment [50]. The comorbid condition of mental illness can increase the risk of exacerbations, reduce QoL, and raise the chance of mortality [70,71]. Thus, mental health illness should receive further piority, which may help to increase QoL among patients living with COPD [72]. 

The results provide some evidence to enhance designing interventions, health research, and policy. Most of the death cases related to COPD happened in LMICs, yet, most of the studies were conducted in HICs. The health research capacity in LMICs is lower than that of HICs could be explained by (1) the limitation of infrastructure and capacity [73], (2) a lack of investment funding in universities and research institutions, low wages for researchers [74], and (3) a lack of clear national research priorities [74]. Therefore, LMICs need to (1) actively create collaboration research networks with HICs and (2) prepare the national research priorities under the circumstance of understanding the local context. Moreover, the LMICs’ national health research priorities should be considered when international organizations or donors from HICs invest in LMICs. Secondly, we call for multidisciplinary collaboration of researchers and physicians among research areas, especially between psychological and respiratory physiologists since the complexity of this disease and negatives effects of depression and anxiety to patients with COPD. 

Several limitations of this study should be mentioned. Firstly, WOS was the only database used in the analysis. However, for a large number of papers for analysis, there was a high possibility that these articles were in other databases, including PubMed and Scopus. Secondly, only English publications were included. Thus, it was more likely that our study did not reflect the trend in COPD research where English is not used. Finally, only titles and abstracts were used for topic modeling. However, by applying a different level of data and alternative method, the trends and hidden themes of the research studies could be discovered [74].

## 5. Conclusions

The findings of the study show that the interventions to increase QoL of patients with COPD has attracted increasing research interest in the last two decades. Non-pharmacological therapy and mental health problems were two common approaches. In addition, increasing support from HICs to LMICs in research together with the multidisciplinary collaboration of research areas are needed to improve the QoL of people living with COPD in LMICs.

## Figures and Tables

**Table 1:** Summary of analytical techniques for each data types.

Type of Data	Unit of Analysis	Analytical Methods	Presentations of Results
Terms, Countries	Words	Frequency of co-occurrence	Map of terms co-occurrence clusters
Abstracts	Papers	Latent Dirichlet Allocation	Ten classifications of research topics
WoS classification of research areas	WoS research areas	Frequency of co-occurrence	Dendrogram of research disciplines (WoS classification)

**Table 2:** General characteristics of publications.

Year Published	Total Number of Papers	Total Citations	Mean Cite Rate per Year	Total Usage Last 6 Month	Total Usage Last 5 Years	Mean Use Rate Last 6 Month	Mean Use Rate Last 5 Year
2018	314	197	0.6	562	822	1.8	0.5
2017	295	1763	3.0	356	1756	1.2	1.2
2016	306	1946	2.1	254	2055	0.8	1.3
2015	270	3311	3.1	219	2198	0.8	1.6
2014	284	5101	3.6	177	2753	0.6	1.9
2013	234	3970	2.8	128	2451	0.5	2.1
2012	199	4653	3.3	78	1825	0.4	1.8
2011	189	6880	4.6	90	1424	0.5	1.5
2010	144	5733	4.4	75	1146	0.5	1.6
2009	128	5719	4.5	61	823	0.5	1.3
2008	127	6549	4.7	36	819	0.3	1.3
2007	109	5158	3.9	33	492	0.3	0.9
2006	107	6800	4.9	28	514	0.3	1.0
2005	86	4625	3.8	37	422	0.4	1.0
2004	75	5445	4.8	30	448	0.4	1.2
2003	62	6317	6.4	20	385	0.3	1.2
2002	56	5847	6.1	15	257	0.3	0.9
2001	40	2757	3.8	3	157	0.1	0.8
2000	43	5670	6.9	28	324	0.7	1.5
1999	28	1612	2.9	6	112	0.2	0.8
1998	32	3324	4.9	14	196	0.4	1.2
1997	29	2223	3.5	8	146	0.3	1.0
1996	18	2275	5.5	3	91	0.2	1.0
1995	16	1681	4.4	9	80	0.6	1.0
1994	10	1060	4.2	0	40	0.0	0.8
1993	12	559	1.8	4	20	0.3	0.3
1992	9	420	1.7	2	14	0.2	0.3
1991	7	426	2.2	0	11	0.0	0.3

**Table 3:** Most cited papers.

No	Title	Total Citation	Published Year	Cite Rate
1	The body-mass index, airflow obstruction, dyspnea, and exercise capacity index in chronic obstructive pulmonary disease	2046	2004	136.4
2	A 4-year trial of tiotropium in chronic obstructive pulmonary disease	1390	2008	126.4
3	Susceptibility to Exacerbation in Chronic Obstructive Pulmonary Disease.	1298	2010	144.2
4	Effect of exacerbation on quality of life in patients with chronic obstructive pulmonary disease	1223	1998	58.2
5	Randomised, double blind, placebo-controlled study of fluticasone propionate in patients with moderate to severe chronic obstructive pulmonary disease: the ISOLDE trial*	964	2000	50.7
6	Development and first validation of the COPD Assessment Test	933	2009	93.3
7	Severe acute exacerbations and mortality in patients with chronic obstructive pulmonary disease	868	2005	62.0
8	Outcomes following acute exacerbation of severe chronic obstructive lung disease	863	1996	37.5
9	Combined salmeterol and fluticasone in the treatment of chronic obstructive pulmonary disease: a randomised controlled trial	749	2003	46.8
10	Chronic obstructive pulmonary disease: current burden and future projections	698	2006	53.7
11	Time course and recovery of exacerbations in patients with chronic obstructive pulmonary disease	655	2000	34.5
12	A long-term evaluation of once-daily inhaled tiotropium in chronic obstructive pulmonary disease	609	2002	35.8
13	Azithromycin for Prevention of Exacerbations of COPD	578	2011	72.3
14	Reduction of hospital utilization in patients with chronic obstructive pulmonary disease—A disease-specific self-management intervention	576	2003	36.0
15	Effects of pulmonary rehabilitation on physiological and psychosocial outcomes in patients with chronic obstructive pulmonary disease	568	1995	23.7
16	Efficacy and safety of budesonide/formoterol in the management of chronic obstructive pulmonary disease	550	2003	34.4
17	Results at 1 year of outpatient multidisciplinary pulmonary rehabilitation: a randomised controlled trial	536	2000	28.2
18	Dyspnea is a better predictor of 5-year survival than airway obstruction in patients with COPD	500	2002	29.4
19	Improved health outcomes in patients with COPD during 1 year’s treatment with tiotropium	492	2002	28.9
20	Maintenance therapy with budesonide and formoterol in chronic obstructive pulmonary disease	472	2003	29.5
21	Global Strategy for the Diagnosis, Management, and Prevention of Chronic Obstructive Lung Disease 2017 Report	467	2017	233.5
22	Relation of sputum inflammatory markers to symptoms and lung function changes in COPD exacerbations	458	2000	24.1
23	Tiotropium in combination with placebo, salmeterol, or fluticasone-salmeterol for treatment of chronic obstructive pulmonary disease—A randomized trial	448	2007	37.3
24	Meta-analysis of respiratory rehabilitation in chronic obstructive pulmonary disease	443	1996	19.3
25	Risk factors of readmission to hospital for a COPD exacerbation: a prospective study	421	2003	26.3
26	Mortality after hospitalization for COPD	380	2002	22.4
27	Depressive symptoms and chronic obstructive pulmonary disease—Effect on mortality, hospital readmission, symptom burden, functional status, and quality of life	369	2007	30.8
28	How well do we care for patients with end stage chronic obstructive pulmonary disease (COPD)? A comparison of palliative care and quality of life in COPD and lung cancer	338	2000	17.8
29	Randomized controlled trial of respiratory rehabilitation	337	1994	13.5
30	Early therapy improves of chronic obstructive outcomes of exacerbations pulmonary disease	335	2004	22.3
31	Quality of life changes in COPD patients treated with salmeterol	331	1997	15.0
32	Prevalence of COPD in Spain: impact of undiagnosed COPD on quality of life and daily life activities	323	2009	32.3
33	A 6-month, placebo-controlled study comparing lung function and health status changes in COPD patients treated with tiotropium or salmeterol	323	2002	19.0
34	Health outcomes following treatment for six months with once daily tiotropium compared with twice daily salmeterol in patients with COPD	317	2003	19.8
35	Roflumilast—an oral anti-inflammatory treatment for chronic obstructive pulmonary disease: a randomised controlled trial	315	2005	22.5
36	Analysis of the factors related to mortality in chronic obstructive pulmonary disease—Role of exercise capacity and health status	314	2003	19.6
37	Interpreting thresholds for a clinically significant change in health status in asthma and COPD	313	2002	18.4
38	Short- and long-term effects of outpatient rehabilitation in patients with chronic obstructive pulmonary disease: A randomized trial	308	2000	16.2
39	Effect of exacerbations on quality of life in patients with chronic obstructive pulmonary disease: a 2 year follow up study	299	2004	19.9
40	Phosphodiesterase-4 inhibitors for asthma and chronic obstructive pulmonary disease	298	2005	21.3

Note: * The inhaled steroids in obstructive lung disease in Europe (ISOLDE).

**Table 4:** Research topics classified by LDA.

Rank by the Highest Volume	Research Topics	*N*	Percent
Topic 2	Pulmonary rehabilitation for COPD	468	16.30%
Topic 1	Comorbidities, mental health and QoL in COPD patients	436	15.20%
Topic 3	QoL of patients with COPD: validity of questionnaire	355	12.40%
Topic 5	Predictors for mortality due to acute exacerbation of COPD	287	10.00%
Topic 7	Pharmacological Therapy and COPD	272	9.50%
Topic 8	Management of COPD	257	9.00%
Topic 4	Multicomponent interventions: home care, rehabilitation, self-care education, integrated care, and pharmacy-led management	255	8.90%
Topic 6	Perception and QoL of patients living with COPD and their caregivers	217	7.60%
Topic 10	Noninvasive Ventilation and Oxygen Therapy in patient with COPD	160	5.60%
Topic 9	COPD Phenotype and quality of life	157	5.50%

## Appendix A

**Table A1:** Search query for “Quality of life” and “well-being”.

No	Search Query	Search Result
# 1	TS = (“quality of life”)	355,541
# 2	TS = (“well-being”)	104,048
# 3	#2 OR #1	441,617
# 4	#2 OR #1	437,253
	Refined by: [excluding] Publication Years: (2019)	
# 5	#2 OR #1	353,171
	Refined by: [excluding] Publication Years: (2019) AND [excluding] Document Types: ( Meeting Abstract Or Proceedings Paper Or Editorial Material Or Book Chapter Or Letter Or Book Review Or Correction Or Note Or News Item Or Book Or Reprint Or Early Access Or Retracted Publication Or Biographical Item Or Correction Addition Or Discussion Or Data Paper Or Retraction Or Bibliography Or Fiction Creative Prose Or Item About An Individual Or Poetry Or Software Review)	
# 6	#2 OR #1	353,170
	Refined by: [excluding] Publication Years: (2019) AND [excluding] Document Types: ( Meeting Abstract Or Proceedings Paper Or Editorial Material Or Book Chapter Or Letter Or Book Review Or Correction Or Note Or News Item Or Book Or Reprint Or Early Access Or Retracted Publication Or Biographical Item Or Correction Addition Or Discussion Or Data Paper Or Retraction Or Bibliography Or Fiction Creative Prose Or Item About An Individual Or Poetry Or Software Review ) AND [excluding] Document Types: (Tv Review Radio Review)	
# 7	#2 OR #1	327,627
	Refined by: [excluding] Publication Years: (2019) AND [excluding] Document Types: (Meeting Abstract Or Proceedings Paper Or Editorial Material Or Book Chapter Or Letter Or Book Review Or Correction Or Note Or News Item Or Book Or Reprint Or Early Access Or Retracted Publication Or Biographical Item Or Correction Addition Or Discussion Or Data Paper Or Retraction Or Bibliography Or Fiction Creative Prose Or Item About An Individual Or Poetry Or Software Review) AND [excluding] Document Types: (Tv Review Radio Review) AND [excluding]Languages: (German Or Spanish Or French Or Portuguese Or Russian Or Turkish Or Polish Or Italian Or Korean Or Czech Or Hungarian Or Croatian Or Greek Or Dutch Or Japanese Or Slovenian Or Slovak Or Lithuanian Or Serbian Or Persian Or Malay Or Romanian Or Chinese Or Icelandic Or Arabic Or Afrikaans Or Norwegian Or Ukrainian Or Danish Or Catalan Or Swedish Or Estonian Or Bulgarian Or Serbo Croatian Or Galician Or Georgian Or Esperanto Or Finnish Or Hebrew Or Indonesian Or Welsh)	
# 8	AU = (“Anonymous” OR “anonymous”)	1,406,800
# 9	#7 NOT #8	327,405
